# Management of Membranous Glomerulonephritis in Pregnancy: A Multidisciplinary Challenge

**DOI:** 10.1155/2015/839376

**Published:** 2015-12-17

**Authors:** Sherifat Ope-Adenuga, Michael Moretti, Nisha Lakhi

**Affiliations:** Richmond University Medical Center, Department of Obstetrics and Gynecology, 355 Bard Avenue, Staten Island, NY 10301, USA

## Abstract

We present a case of 28-year-old female, who had a past obstetrical history complicated by uncontrolled blood pressure, early onset preeclampsia, and a fetal demise at 29 weeks. Her blood pressure normalized after each pregnancy, and no diagnosis of renal disease was ever established. In her most recent pregnancy, she remained normotensive and initially presented with normal blood urea nitrogen and creatinine levels. However, after the early first trimester, she developed nephrotic range proteinuria, hypoalbuminemia, and peripheral edema. After delivery of the baby, all clinical symptoms rapidly resolved and laboratory values normalized. We review the clinical course, diagnosis, and management of new onset nephrotic syndrome in pregnancy.

## 1. Introduction

Even before conception occurs, adaptive renal changes for a possible pregnancy commence. During the luteal phase of each menstrual cycle, renal blood flow and glomerular filtration rate (GFR) increase by 10–20% [1]. If pregnancy is established, these hemodynamic changes continue. By the midsecond trimester, renal blood flow peaks to 70–80% above nonpregnant levels, leading to an increase in GFR of approximately 55% [1]. The effect of pregnancy and its associated physiological adaptive changes can unmask occult underlying renal disease with proteinuria. In addition, the presence underlying glomerular disease can lead to increased pregnancy complications and have adverse effect on fetal outcome. Although proteinuria can be a normal finding during pregnancy, it represents underlying renal disease if present before 16-week gestation [1]. Nephrotic range proteinuria should not occur and is considered pathological at any trimester of pregnancy [1].

## 2. Case Report

A 28-year-old, Jamaican female, gravida 7 para 3 presented to the clinic at 8-week gestation for her first prenatal visit. Her first pregnancy was complicated with uncontrolled hypertension resulting in a term primary cesarean delivery. Prior to this pregnancy she was normotensive. As this pregnancy occurred in Jamaica, medical records regarding the details of her care were not available. The indication for the cesarean delivery was not known to the patient. However, the patient reported that her blood pressure normalized after this delivery. This was followed by an uncomplicated pregnancy and repeat cesarean delivery at term three years later. Over the next three years, she had three elective terminations of pregnancy. A year thereafter, she had another pregnancy that was complicated by proteinuria and elevated blood pressure that resulted in a fetal demise at 29-week gestation. After delivery, her blood pressure normalized and she remained asymptomatic. The presumptive diagnosis by the medical team was early onset severe preeclampsia, and therefore no renal biopsy or subsequent workup was undertaken at the time.

**Figure 1:**
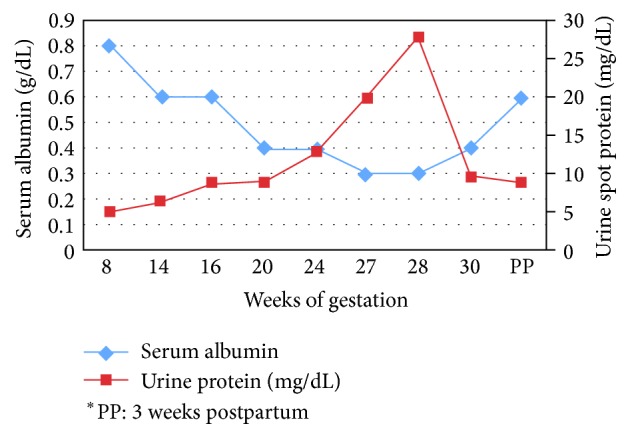
Graph of serum albumin and protein versus gestational week of pregnancy.

A year after the last pregnancy she emigrated to the United States and presented to our clinic for prenatal care at 8-week gestation. She denied any medical problems and was not using any medications. She smoked one pack of cigarettes per day and denied the use of alcohol or other illicit drugs. Her initial blood pressure was 96/60 mmHg. Prenatal labs, initial complete blood count, and BUN and creatinine levels were normal (Figure 1 and Table 1).

At 12-week gestation, she returned to clinic for follow-up. Physical examination was positive for 1+ bilateral lower extremity edema. Her blood pressure was 102/64 mmHg. A baseline 24-hour urine collection revealed 5047 g of protein excretion. Subsequent workup with renal ultrasound, microscopic urine analysis, urine electrolytes, and a rheumatology panel consisting of anti-nuclear antibody (ANA), CRP, anti-double stranded DNA, anti-JO-1 antibody, Sjogren's antibody, anti-DNA antibody, anti-cardiolipin antibody, complement C3 and complement C4, thyroid antibody, and anti-smooth muscle antibody was undertaken.

By 13-week gestation, the patient gained five pounds over one week, her bilateral lower extremity edema increased 2+, and she now complained of mild shortness of breath. Blood pressure was 110/60. Her rheumatology workup was negative, except for ANA which was positive with a nucleolar pattern. She was started on a sodium restricted diet and jointly managed with a nephrologist and rheumatologist.

Follow-up at 15-week gestation showed worsening of her lower extremity edema and another five-pound weight gain. She was continued on a sodium restricted diet (2000 mg daily) and started on furosemide 20 mg daily. A week later, there was no improvement in her edema and an additional five-pound weight gain was noted. The dose of furosemide was increased to 40 mg daily. Laboratory results at this time revealed the following: 24 hr urine protein 8020 g, BUN 10 mg/dL, creatinine 0.4 mg/dL, and albumin 0.8 mg/dL.

Between 16-week gestation and 23-week gestation the patient was closely monitored with weekly weights, urine protein collection, and blood pressure and for fetal wellbeing. Based on the worsening of her proteinuria range (11 g/day–18 g/day), severe hypoalbuminemia, and bilateral lower extremity edema, she was diagnosed with nephrotic syndrome.

At 24-week gestation, patient presented to the labor and delivery triage unit. Examination revealed +4 bilateral lower extremity edema with fluid tracking up to the abdomen. The patient stated that she was unable to ambulate. Her 24-urine protein had increased to 13,000 g. Her BUN was 7 mg/dL, creatinine was 0.7 mg/dL, and blood pressure was 111/67 mmHg. The patient was admitted to the hospital and subsequently started on 25% intravenous albumin and 40 mg furosemide twice a day for diuresis.

**Figure 2:**
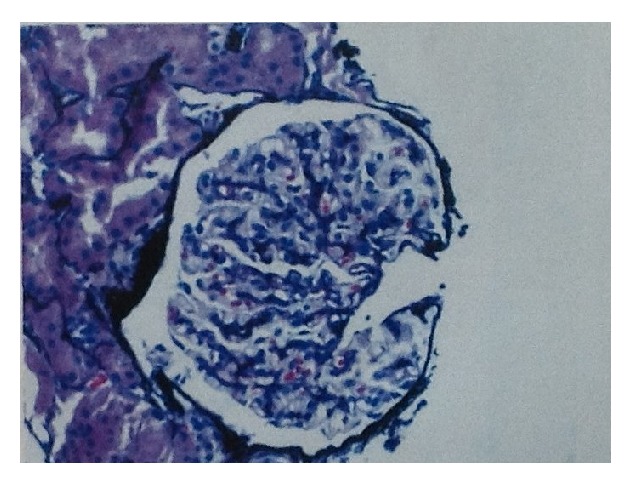
Light microscopy with thickened capillary loops.

**Figure 3:**
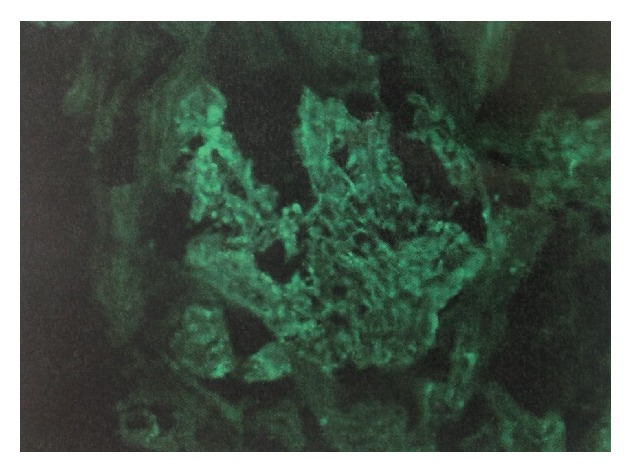
Direct immunofluorescence showing granular staining in capillary loops for IgG.

**Figure 4:**
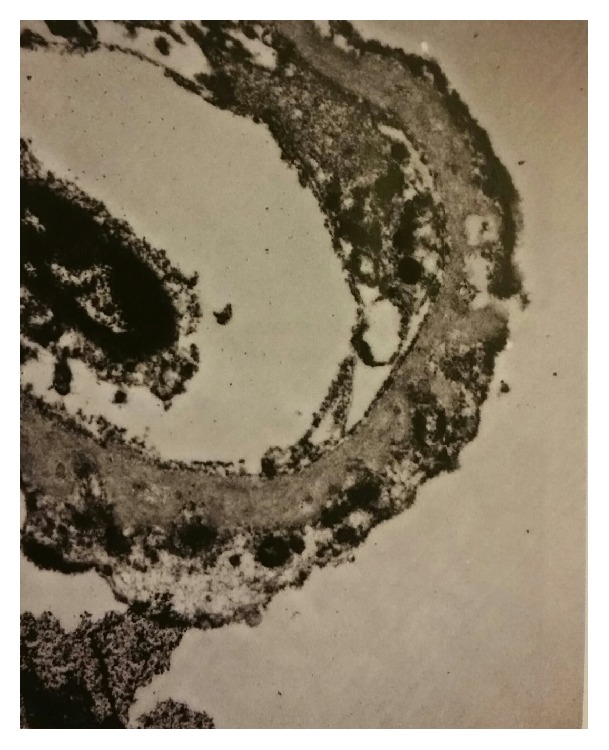
Electron microscopy with global thickening of the glomerular basement membrane.

Renal biopsy was done at 25 weeks. On light microscopy (Figure 2), the glomeruli were enlarged, and 3/17 glomeruli were globally sclerosed. The mesangium showed an increase in cellularity. There was no endocapillary proliferation, crescents, or fibrinoid necrosis present. The capillary loops were thickened and showed spikes on sliver methenamine stain. The tubules showed focal signs of acute tubular injury with vacuolation, blebbing, dilatation, and nuclear dropout. There was not vasculitis or vascular necrosis present. PLA2R was negative. On direct immunofluorescence (Figure 3) there was granular staining in capillary loops for IgG (2+), IgA (1+), IgM (trace), C3 (trace), C1q (trace), kappa (1+), lambda (2+), and fibrinogen (trace). There was no significant glomerular staining for albumin, nor significant staining in the tubular basement membrane or vessel walls. Electron microscopy (Figure 4) revealed global thickening of the glomerular basement membrane due to subepithelial and intramembranous immune type electron dense deposits. There were no subendothelial or mesangial immune type deposits noted.

The patient was started on Tacrolimus 2 mg daily and 10 mg oral prednisone at 27 weeks. She lost 5 pounds in the following two weeks. Fetal growth continued to be appropriate for gestational age and she denied any headache, visual disturbances, or elevated blood pressure.

She represented in preterm labor at 30-week gestation. Her vital signs were stable with BP: 110/77, HR: 80, and proteinuria of 18 g. She subsequently delivered a 2 lbs 8 oz female infant with Apgar scores of 3 and 6 at 1 and 5 minutes, respectively. The patient symptoms improved significantly after delivery. She had lost 70 pounds of fluid weight by 3 weeks postpartum and continued on the regimen of Tacrolimus and prednisone.

## 3. Discussion

Membranous glomerulonephritis, a cause of nephrotic syndrome, is histopathologically defined by the presence of immune complexes on the extracapillary side of the glomerular basement membrane [2]. Most often this condition is idiopathic; however, it can be secondary to wide spectrum of infections, tumors, autoimmune diseases, or exposure to drugs or toxic agents [2].

Due to the hemodynamic changes associated with pregnancy, renal disease may initially be masked. The increase in GFR during pregnancy leads to a fall in serum creatinine concentration, so that values that are normal in the nonpregnant state may be considered elevated during pregnancy. Proteinuria increases as pregnancy progresses while serum albumin levels decline by 5–10 g/L. However, the presence of nephrotic range proteinuria with or without hypertension in the first trimester is pathological and may be associated underlying renal disease and a poor prognosis [3]. In our patient's reported pregnancy, her renal condition gradually deteriorated from the first trimester, with worsening proteinuria, hypoalbuminemia, and peripheral edema. In patients presenting with significant proteinuria during early gestation, biopsy is necessary as treatment options differ depending on the etiological cause.

Although some studies have shown good neonatal outcomes in patients with nephrotic syndrome [4], others have demonstrated rates of fetal loss ranging from 24 to 35% [5–7]. Most of these losses were attributed to first trimester spontaneous abortions. In a systematic review of six studies, Lindheimer and Katz concluded that the average live birth rate in patients with membranous glomerulonephritis was 86.3%, with 4% of the losses occurring after the first trimester [7]. This data is in agreement with a study by Jungers et al. that retrospectively reviewed 43 pregnancies associated with impaired renal function. Of the 43 pregnancies, 13 ended in fetal death (including 5 first-trimester abortions and 8 fetal deaths beyond the 20th gestational week) [6]. Other adverse fetal outcomes that have been associated with nephrotic syndrome include preterm delivery and low birth weight; however, results for these outcomes have not been consistent between studies [5].  Table 2 summarizes pregnancy courses for reported cases of biopsy proven membranous glomerulonephritis.

The patient's obstetric history was significant for multiple adverse outcomes. In her first pregnancy, she suffered from gestational hypertension and delivered a term infant in Jamaica by primary cesarean section. The exact indication for cesarean delivery is unclear, as medical records from Jamaica were not available. However, as per the patient's history, it was done urgently due to elevated blood pressure. She then had a period of three to four years of disease remission, where she had a normal pregnancy. Subsequently, she suffered a fetal loss at 29-week gestation. That pregnancy was also complicated by proteinuria and hypertension. It is quite possible that she had membranous glomerulonephritis during that pregnancy, and the presumptive diagnosis of early onset preeclampsia was incorrect. A renal biopsy at that time could have clarified the diagnosis. The severity of symptoms can vary with different pregnancies and may be dependent on underlying renal function around the time of conception [3]. Overt nephrotic syndrome and hypertension at the onset of gestation are associated with a worse prognosis. Therefore, her prepregnancy renal function could have influenced the course of her prior pregnancies accounting for the variability in outcomes.

Managing nephrotic syndrome in pregnancy is difficult. Our patient presented with significant weight gain secondary to peripheral edema. The patient's intravascular fluid status, as opposed to the severity of peripheral edema, needs to be assessed when administering diuretic therapy. Many patients with a low serum albumin may have gross peripheral edema but may have diminished intravascular volume. Aggressive diuresis will worsen the intravascular depletion, causing poor placental perfusion and increasing the risk of acute renal failure [1].

Adequate anticoagulation in pregnant patients with nephrotic range proteinuria is important, as renal vein thrombosis has been reported [8]. Nephrotic syndrome is associated with hypercoagulability due to increased clotting factors V, VII, and VIII, fibrinogen, and 2-antiplasmin and depletion of factors IX and XII, antithrombin III, and plasminogen. Adaptations of pregnancy, including increased fibrinogen, factors VII, VIII, and X, and decreased fibrinolytic activity, also increase hypercoagulability [8].

In order to optimize both maternal and fetal outcomes in patients with known renal disease, preconceptional counselling is essential. Malik et al. retrospectively reported outcomes of repeated pregnancies in patients with known primary membranous [5]. Of the 30 pregnancies, there was a 90% live birth rate with only one perinatal mortality reported [5]. Optimization of the both maternal renal status and hypertension before attempted pregnancy improves outcomes. Jungers et al. demonstrated higher live birth rates in pregnancies that started with serum creatinine levels less 0.20 mmol/L than in those with serum creatinine greater than 0.20 mmol/L (80% versus 53%, *p* = 0.02). The presence of maternal hypertension was the major factor influencing fetal prognosis, as the relative risk of fetal loss was 10.6 times higher when hypertension was present at conception or early in pregnancy compared to when blood pressure was normal or well-controlled by therapy [6]. In patients that had both uncontrolled hypertension and proteinuria at conception, an accelerated course toward end-stage renal failure was observed in 7 patients (23%) [6]. Therefore appropriate timing of pregnancy and optimization of both maternal blood pressure and renal function can allow better outcomes.

## Figures and Tables

**Table 1:** Laboratory values, body weight, and blood pressure during pregnancy.

Gestational Age	8	14	16	20	24	27	28	30	PP
Hemoglobin (g/dL)	12.1	10.9	10.2	10.8	10.1	10.6	10.3	10.4	10.6
Hematocrit (%)	37.1	33.7	31.4	32.9	31.9	33.4	32.7	31.5	33.6
Platelets (k/*μ*L)	236	227	247	263	323	271	359	254	267
Albumin (g/dL)	0.8	0.6	0.6	0.4	0.4	0.3	0.3	0.4	0.6
BUN (mg/dL)	7	10	9	9	9	10	12	12	14
Creatinine (mg/dL)	0.3	0.2	0.6	0.6	0.6	0.7	0.6	0.6	0.6
Urine spot protein (mg/dL)	5	6.3	8.8	9.0	13.0	20.0	28.0	9.6	9.0
Body weight (pounds)	145	155	161	175	203	195	200	210	215
Blood pressure (mmHg)	96/60	100/60	100/62	102/64	110/70	100/54	100/68	105/68	103/69

**Table 2:** Case reports of membranous glomerulonephritis in pregnancy.

Author	Disease history	Treatment	Maternal outcome	Fetal outcome
Katzir et al. [3], 2004	23 y/o with known MGN	Methylprednisolone pulse therapy Oral prednisolone	Proteinuria, HTN Preeclampsia at 34 weeks HTN, proteinuria resolved following delivery	C/S at 34 weeks, secondary failed induction for preeclampsia Male, 2,090 g

Sebestyen et al. [9], 2008	33 y/o with known MGN Previous pregnancy with preeclampsia at 36 weeks	Methylprednisolone pulse therapy Oral prednisolone Azathioprine	Deterioration of creatinine clearance, low serum total protein, increasing edema Three months after delivery, maternal condition went into complete remission	IUGR C/S at 33 weeks due to IUGR and deterioration of maternal renal function Male, 1,160 g

Aoshima et al. [10], 2013	37 y/o, no history of MGN Previous normal pregnancy	Methylprednisolone pulse therapy Oral prednisolone	Increasing edema Increasing proteinuria 5.28 g/day Diagnosed with MGN at after pregnancy termination Symptoms resolved after termination	Elective termination due to worsening symptoms at 21 weeks

Ope-Adenuga et al., index patient	28 y/o, no history of MGN First pregnancy: uncontrolled HTN Second pregnancy: uncomplicated Third pregnancy: possible preeclampsia, fetal demise at 29 weeks Fourth pregnancy: index case	Tacrolimus Oral prednisone	Increasing edema, worsening of renal function, increased proteinuria Diagnosed with MGN at 25-week gestation Symptoms improved during treatment and completely resolved after delivery	Preterm labor at 30 weeks Female, 1021 g
